# Uterine histomorphological and immunohistochemical investigation during the follicular phase of estrous cycle in Saidi sheep

**DOI:** 10.1186/s12917-024-04456-3

**Published:** 2025-01-13

**Authors:** Mahmoud Abd-Elkareem, Mohsen A. Khormi, Mohammed A. Alfattah, Mervat S. Hassan

**Affiliations:** 1 Department of Cell and Tissues, Faculty of Veterinary Medicine, Assiut University, Assiut, 71526 Egypt; 2 Department of Biology, College of Science, Jazan University, P.O. Box. 114, Jazan, 45142 Kingdom of Saudi Arabia; 3 Theriogenology Department, Faculty of Veterinary Medicine, New-Valley University, New Valley, 725211 Egypt

**Keywords:** Saidi sheep, Uterus, Endometrium, Estrous cycle, Oxidative stress, Progesterone receptors, Mast cells

## Abstract

**Background:**

Saidi sheep are one of the most important farm animals in Upper Egypt, particularly in the Assiut governorate. Since they can provide meat, milk, fiber, and skins from low-quality roughages, sheep are among the most economically valuable animals bred for food in Egypt. Regarding breeding, relatively little is known about the Saidi breed. In mammals, the uterus is a crucial reproductive organ. Therefore, the purpose of this work was to provide further details on the histological, histochemical, and immunohistochemical analyses of superoxide dismutase 2 (SOD2), glutathione reductase (GR), and progesterone receptor alpha (PRA) as well as terminal deoxynucleotidyl transferase (TdT) dUTP nick-end labeling assay (TUNEL) of the uterus during the follicular phase of estrous cycle in Saidi sheep. Thus, 11 healthy Saidi ewes (38.5 ± 2.03 kg weight) ranging in age from 2 to 5 years were used to examine the histological changes in the uterus.

**Results:**

In Saidi sheep, the uterine histological and immunological picture during the follicular phase of the estrous cycle was characterized by epithelial and stromal proliferation and apoptosis. Leucocytic recruitment (lymphocytes, plasma, and mast cells) was also observed. Uterine gland adenogenesis, vascular angiogenesis, oxidative marker expression, and PRA expression in the muscles, stroma, and epithelium were the most noticeable features of the follicular phase.

**Conclusion:**

This study provides new evidence of the role of PRA, SOD2, GR, and mast cells in controlling uterine epithelial proliferation and apoptosis in the Saidi sheep during the follicular phase of the estrus cycle. These findings have growing significance in understanding the key mechanisms that characterize successful reproduction and enhancing the fertility and reproductive efficiency in Saidi Sheep.

## Background

Sheep are extensively raised as livestock worldwide because they yield a lot of meat, fat, milk, wool, and other useful products. Many studies have been focused on improving sheep reproductive performance and litter size [1]. The Saidi breed of Egyptian sheep was once thought to be the oldest breed of Egyptian sheep, and its breeding grounds are in Upper Egypt, south of Assiut. It has a long, thick tail and its fleece is typically dark brown [2]. Due to its high reproductive performance, the demand for this breed increases [3– 5]. Despite Saidi ewes having almost no seasonal variation in their reproductive cycles, estrous activity decreases in the spring [6].

The uterus is an important organ for reproduction in mammals. The uterus provides a required microenvironment for the following processes: (1) transportation, storage, and maturation of spermatozoa; (2) synthesis of prostaglandin F2α, the luteolysin required for ovarian cyclicity in domestic animals; (3) offering of an embryotrophic environment for conceptus (embryo/fetus and associated extraembryonic membranes) growth and development; and (4) pickup of the conceptus at parturition [7– 9]. The uterus of sheep is bicornuate, with a single cervix and a small common corpus. Histologically, the uterus in ewe formed of endometrium, myometrium, and perimetrium. The main cyclic changes occurred in the endometrium and a lesser extent in the myometrium [10– 12].

The endometrium of all mammalian uteri contains glands that produce, transport, and release chemicals necessary for survival and development of the conceptus (the embryo/fetus and related extraembryonic tissues). Adult ruminants’ endometrium is composed of several aglandular caruncular regions and intercaruncular areas, each of which contained hundreds of glands per uterine wall cross-Sects. [10, 13, 14]. In all mammals, uterine glands and the secretory products they produce are probably important regulators of uterine receptivity, blastocyst implantation, stromal cell decidualization and growth and development of the conceptus throughout the first trimester [13, 15].

The myometrium was formed of a thick, inner circular layer and a thin, outer longitudinal layer of smooth muscle fibers. A well vascularized stratum vascularis with many branches of the uterine artery was observed in the outer most layer of inner circular myometrium [10, 16].

The uterine arteries are the main blood supply to the uterus. They were found inside the myometrium. During the proliferative phase, the subepithelial capillary plexus has the highest vascular length density. Endothelial proliferation is the main mechanism of endometrial angiogenesis during the proliferative phase and is influenced by estrogen. Estradiol stimulates vascular permeability, angiogenesis, and endothelial cell proliferation in response to VEGF [17]. The estrous cycle is regulated in large part by the uterine blood supply. The two main hormones influencing blood flow in the arteries, which feed the uterus, are estrogens and progesterone. Vasodilation and vasoconstriction are regulated, complemented, and supported by the following factors: PGE2, LH, oxytocin, cytokines, neurotransmitters, and other local blood flow regulators [18].

Progesterone, via binding to PR-A regulates the development and function of the endometrium and stimulates myometrial relaxation and cervical closure [19, 20]. Remarkably, during the follicular phase of the estrous cycle, mast cells (MCs) are essential for secreting chemicals that support tissue remodeling [21]. Many physiologically active chemicals are synthesized and released by MCs; some of these compounds are preformed and kept in their granules for quick release. (histamine, TNF-α, heparin, lysosomal hydrolases, and proteases) [22]. There was an inverse relationship between apoptosis and cell proliferation in uterine and vaginal epithelial cells during the estrous cycle [23, 24]. It was found that SOD2 and GR expression help to control apoptosis [5] and other oxidative stress [25, 26].

The key mechanisms that characterize successful reproduction and gaps in knowledge must be the subject of the research to enhance the fertility and reproductive health of Saidi Sheep, mainly when little is known about the uterine picture of the Saidi sheep during the follicular phase of the estrous cycle. Therefore, this study aims to give more details on the histological, histochemical, and immunohistochemical analyses (SOD2, RG, PRA, TUNEL assay, and mast cell tryptase) of the uterus during the follicular phase of estrous cycle in Saidi sheep.

## Materials and methods

### Animals and samples

Uteri were collected from eleven healthy Saidi sheep aged 2 to 5 years and weighed (38.5 ± 2.03 Kg) after slaughter (according to Islamic religion) at Assiut slaughterhouse, Assiut governorate, Egypt.

## Histological examination

Uteri (*n* = 11) were fixed in 10% neutral buffered formalin then thoroughly washed and dehydrated in ascending series of ethanol. The dehydrated samples were cleared in methyl benzoate and infiltrated with paraplast at 58–60 °C followed by blocking out. Paraffin sections at 3–5 μm in thickness were cut and stained with the following histological stains:

- Haematoxylin and Eosin for general histological examination of the uterus [27].
- Periodic acid Schiff (PAS) technique for demonstration of glycoprotein [27].
- Masson’s trichrome technique for staining collagen fibers [28].
- Picro-Sirius red technique for differentiation between mature and immature collagen fibers in the uterus [29, 30].
- Orcien stain for detection of the distribution of elastic fibers in the uterus [31].

## Oxidative stress detection

**Immunohistochemistry of glutathione reductase (GR) and superoxide dismutase 2 (SOD2).**

Uterine tissue sections were deparaffinized, rehydrated, and washed in phosphate-buffered saline (PBS). Slides were then submerged in 10 mM sodium citrate buffer (pH 6.0) for 20 min at 95–98 °C in a water bath to retrieve antigens. The slides were incubated in 3% hydrogen peroxide for 10 min at room temperature to suppress endogenous peroxidase. The slides were then cleaned in PBS three times, for five minutes each. Rabbit polyclonal antibodies were incubated with the sections for a whole night in a humid chamber. We utilized polyclonal anti-glutathione reductase and anti-superoxide dismutase 2 antibodies, for the immunohistochemical detection of GR and SOD2 respectively in the uterus (Chongqing Biospes Co., Ltd, China) and Power-Stain™ 1.0 Poly horseradish peroxidase (HRP) DAB Kit (Genemed Biotechnologies, Inc, 458 Carlton Ct. South San Francisco, CA 94080, USA) [32].

## Apoptosis detection

The In Situ Cell Death Detection Kit, Fluorescein (Sigma-Aldrich, USA) was used to detect apoptosis. The TUNEL assay; terminal deoxynucleotidyl transferase (TdT) dUTP Nick-End Labeling, was developed to identify apoptotic cells that display significant DNA fragmentation in the final stages of their death. This technique relied on the capacity of TdT to mark double-stranded DNA break blunt ends without using a template according to the method previously published [33]. After rinsing the slides in PBS, they were examined under a fluorescent microscope.

### Immunohistochemical detection of progesterone receptor alpha (PRA) and mast cells (MCs)

The technique was applied according to the company’s recommendations and our lab protocol [34]. The fixed uteri underwent ethanol dehydration, methyl benzoate clearing, and paraplast embedding. Xylene was used to dewax paraplast embedded tissue Sect. (5 μm). After rehydrating with 100%, 95%, 80%, and 70% ethanol, the slides were rinsed with PBS (pH 7.4). Endogenous peroxidase was inhibited by 3% hydrogen peroxide and then washed in PBS. The slides were cooled to room temperature after being submerged in 10 mM sodium citrate buffer (pH 6.0) at 95–98 °C for 20 min to detect antigens. After that, sections were cleaned in PBS. Then, sections were incubated at room temperature for 30 to 60 min with the primary antibodies. Immunolocalization of PRA was done by using; progesterone receptor rabbit pAb (Catalog No.: A0321), ABclonal, USA. On the other hand, we employed Mast Cell Tryptase (3G3) Monoclonal Antibody (Bioss antibodies) to identify MCs. The slides were washed with PBS and subjected to a two-step poly Q stain detection system goat anti-mouse/rabbit HRP, peroxidase quench, and DAB kit, Quartett, Germany. Harris hematoxylin was used as a counterstain on the sections for 30 s. Sections were then dehydrated with 95% and 100% alcohol, cleared in xylene, and mounted with DPX.

An Olympus BX51 microscope was used to examine each staining slide, and an Olympus DP72 camera was used to take pictures.

## Results

### General uterine histomorphological characters during the follicular phase of the estrous cycle in Saidi sheep

**Fig. 1:**
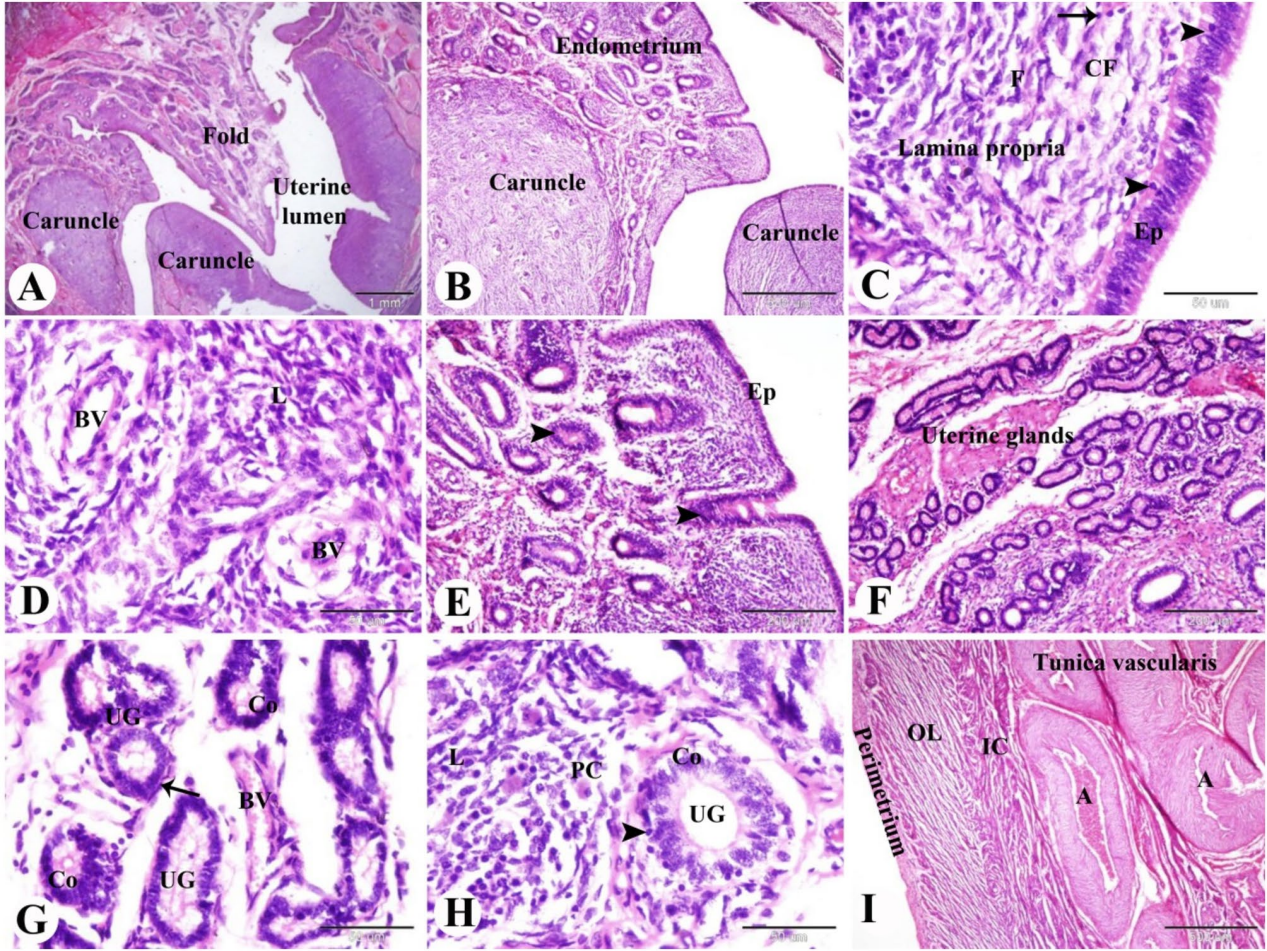
Photomicrograph of the sheep uterus during the follicular phase of the estrous cycle. (**A & B**): Showing the endometrium with mucosal folds, caruncles, and narrow uterine lumen. (**C**): Showing the endometrium formed of lamina epithelialis (EP) of pseudostratified columnar epithelium with intraepithelial lymphocytes (arrowheads) and connective tissue lamina propria contained fibroblasts (F), collagen fibers (CF) and lymphocytes (arrow). (**D**): lamina propria contained many blood vessels (BV) and leucocytic infiltrations (L). (**E**): Showing many epithelial (EP) invaginations (arrowheads) which form the uterine glands (arrowhead). (**F & G**): Showing lamina propria with blood vessels (BV) and highly branched and highly coiled uterine glands (UG) which formed of columnar epithelium (Co) and surrounded by myoepithelial cells (arrow). (**H**): Showing intraepithelial lymphocytes (arrowheads) between the columnar cells (Co) of the uterine glands (UG), plasma cells (PC) and leucocytic infiltration (L) in lamina propria. (**I**): Showing well vascularized tunica vascularis with many branches of the uterine artery (A) in the outer most layer of inner circular myometrium (IC). Note the outer longitudinal (OL) muscle fibers of myometrium and perimetrium. Original magnification; A: X12.5, scale bar = 1 mm, B & I: X40, scale bar = 500 μm, C, D, G & H: X400, scale bar = 50 μm, E & F: X100, scale bar = 200 μm, haematoxylin and eosin stain

Microscopical analysis of the uterus of Saidi sheep during the follicular phase of the estrous cycle revealed that the uterine wall was composed of three layers: the inner layer; endometrium (mucosa), the middle layer; myometrium (muscular layer), and the outer layer; perimetrium (serosa). The endometrium had mucosal folds, caruncles, and narrow uterine lumen. The caruncles were non glandular, highly cellular, and highly vascular endometrial elevation or projections. The endometrium formed of lamina epithelialis of pseudostratified columnar epithelium with intraepithelial lymphocytes and connective tissue lamina propria contained uterine glands, fibroblasts, collagen fibers, blood vessels and leucocytic infiltrations especially lymphocytes and plasma cells (Fig. 1A-D).

The current study showed that the endometrium in Saidi sheep during the follicular phase of the estrous cycle was characterized by epithelial proliferation and many epithelial invaginations which form the uterine glands (uterine gland adenogenesis). The uterine glands were highly branched and highly coiled and formed of columnar epithelium which is surrounded by myoepithelial cells and had intraepithelial lymphocytes (Fig. 1E-H).

The myometrium is form of a thick, inner circular layer, and a thin, outer longitudinal layer of smooth muscle fibers. A well vascularized stratum vascularis with many branches of the uterine artery was observed in the outer most layer of the inner circular myometrium. The outer perimetrium was formed of mesothelial layer, and submesothelial connective tissue (Fig. 1I).

### Glycoprotein localization in the Saidi sheep uterus during follicular phase of estrous cycle

**Fig. 2:**
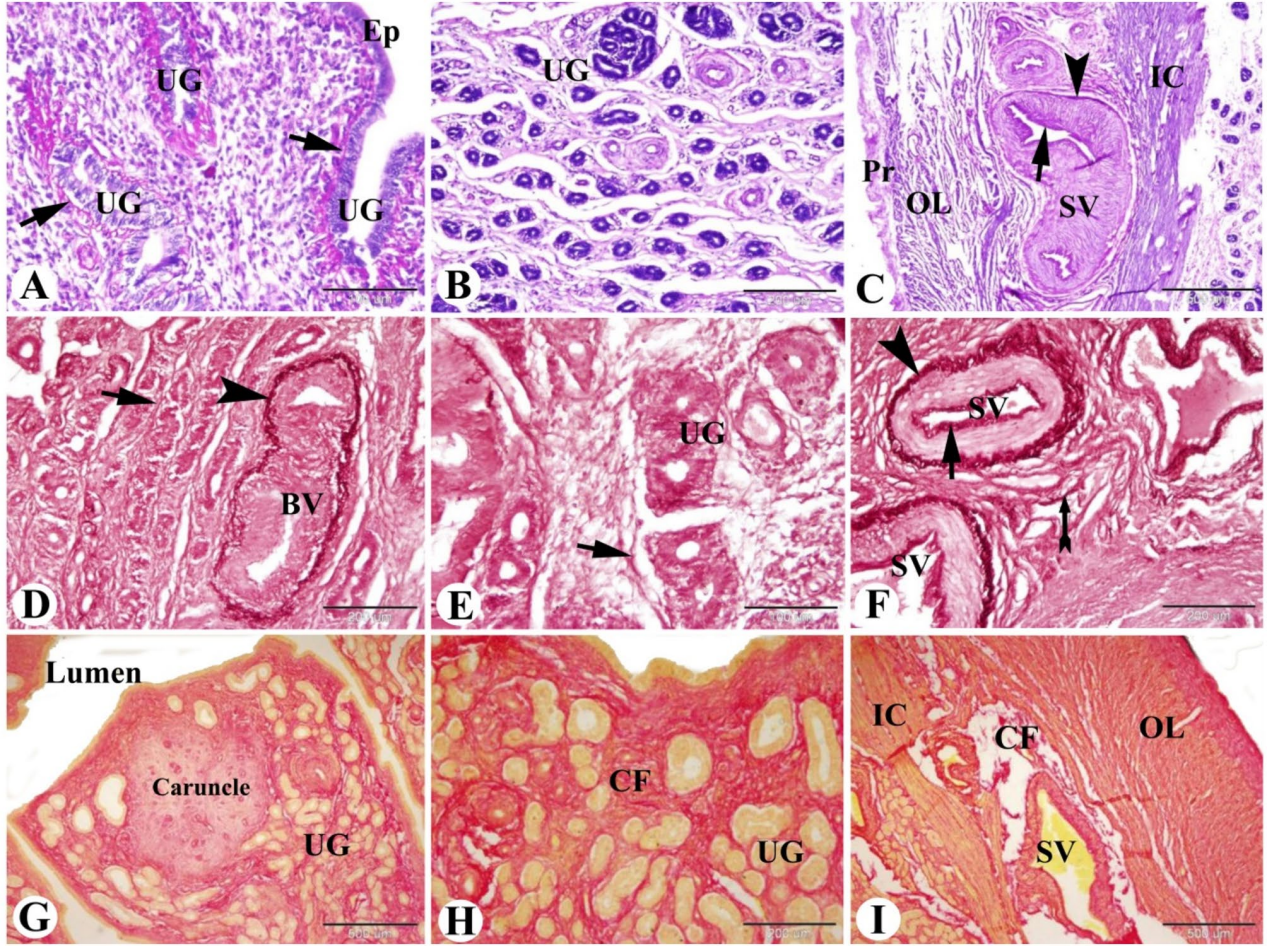
Photomicrograph of the sheep uterus during the follicular phase of the estrous cycle. (**A & B**): Showing the endometrium with PAS positive epithelial basement membrane (arrows) of lamina epithelialis (Ep) and uterine glands (UG). Note no PAS positive secretion in the uterine glands. (**C**): Showing well-vascularized tunica vascularis with PAS positive internal (arrow) and external (arrowhead) elastic lamina in the outer most layer of inner circular myometrium (IC). Note the outer longitudinal (OL) muscle fibers of the myometrium and the perimetrium (Pr). (**D & E**): Showing the endometrium in the subepithelial and between the uterine glands (UG) with few elastic fibers (arrow) except for blood vessels; external elastic lamina (arrowhead) of endometrial blood vessels (BV). (**F**): Showing the myometrium with few elastic fibers between the smooth muscle fibers (forked tail arrow) except for blood vessels of stratum vascularis; in the internal (arrow) and external (arrowhead) elastic lamina. (**G & H**): Showing the endometrium with a moderate amount of collagen fibers (red color, CF) between the uterine glands (UG) and a few amounts in the caruncle. **I**: Showing the myometrium with few amount of collagen fibers (red color) in between the smooth muscle fibers of the inner circular (IC) and outer longitudinal (OL) layers. Note the moderate amount of collagen fibers (CF) around blood vessels in the stratum vascularis (SV). Original magnification; A: X200, scale bar = 100 μm, B: X100, scale bar = 200 μm, C: X40, scale bar = 500 μm, PAS & Hx. D: X200, scale bar = 100 μm, E: X100, scale bar = 200 μm, F: X200, scale bar = 100 μm, Orcien stain. G: X40, scale bar = 500 μm, H: X100, scale bar = 200 μm, I: X40, scale bar = 500 μm, Picro-Sirius red technique

Glycoprotein localization in the sheep uterus during the follicular phase of the estrous cycle revealed that the endometrium had a PAS positive epithelial basement membrane of lamina epithelialis and uterine glands. No PAS positive secretion in the uterine glands could be observed. PAS positive internal and external elastic lamina in the stratum vascularis could be detected (Fig. 2A-C).

### Elastic and collagen fibers distribution in the Saidi sheep uterus during the follicular phase of estrous cycle

Elastic fiber distribution in the Saidi sheep uterus during the follicular phase of the estrous cycle revealed few elastic fibers were observed in the subepithelial and between the uterine glands in the endometrium. Furthermore, few elastic fibers between the smooth muscle fibers in the myometrium were observed. However many elastic membranes were observed in the external elastic lamina of endometrial blood vessels, and in the internal and external elastic lamina of the blood vessels of the tunica vascularis (Fig. 2D-F).

**Fig. 3:**
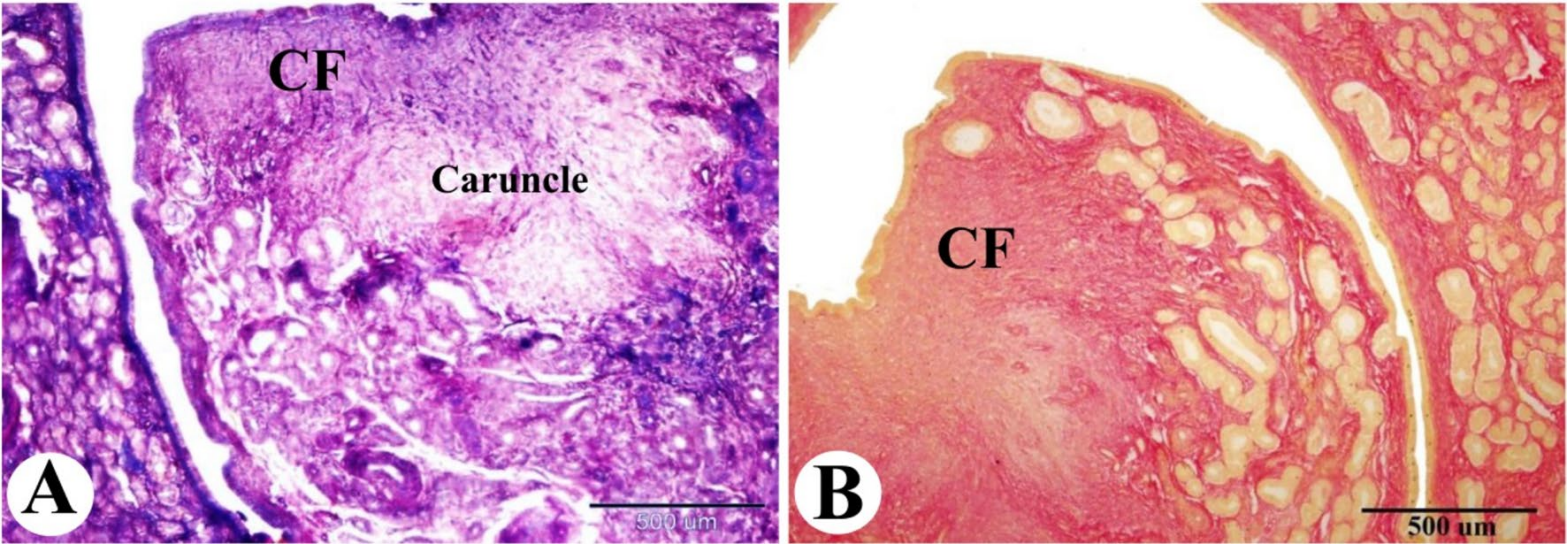
Photomicrograph illustrating the collagen fiber distributions in the sheep uterus during the follicular phase of the estrous cycle. (**A & B**): Showing the lamina propria of the endometrium with a moderate amount of collagen fibers (blue color with Masson’s trichrome technique and red color with Picro-Sirius red technique, CF) between the uterine glands (UG) and few amounts in the caruncle. Original magnification; (**A & B**): X40, scale bar = 500 μm, (**A**): Masson’s trichrome technique, (**B**): Picro-Sirius red technique

The collagen fibers distribution in the Saidi sheep uterus during the follicular phase of the estrous cycle revealed that the endometrium had a moderate amount of collagen fibers between the uterine glands and a few amounts in the caruncles. Whereas the myometrium had few amounts of collagen fibers in-between the smooth muscle fibers in the inner circular and outer longitudinal layers, and a moderate amount of collagen fibers around blood vessels in the stratum vascularis (Figs. 2G-I and 3A, amp and B).

### GR and SOD2 immunolocalization in the Saidi sheep uterus during the follicular phase of the estrous cycle

**Fig. 4:**
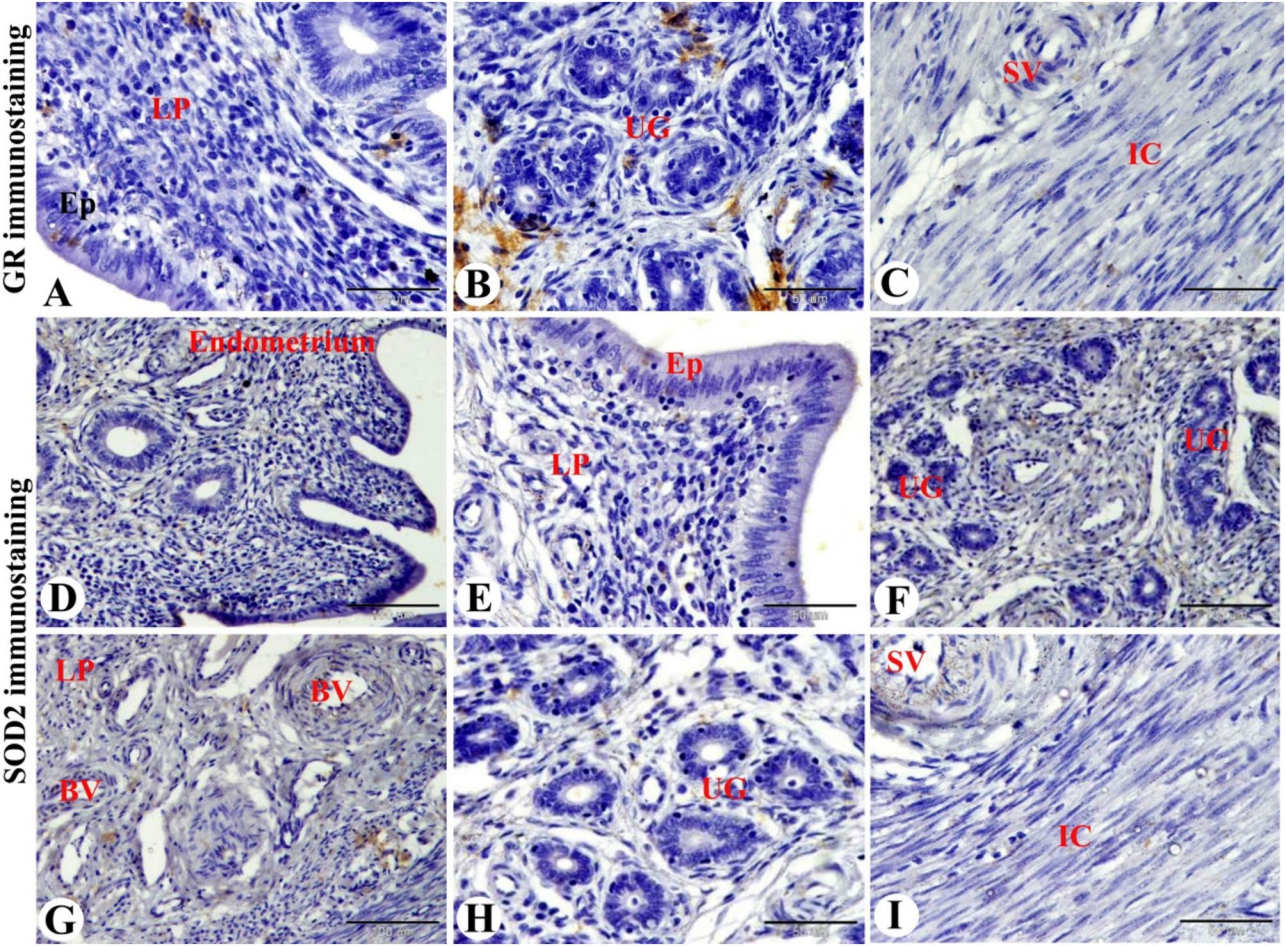
Photomicrograph of GR immunostaining in the sheep uterus during the follicular phase of the estrous cycle. (**A & B**): Showing slight GR immunostaining in the lamina epithelialis (Ep), in the stroma cells of lamina propria (LP), and negative GR immunostaining in the uterine glands (UG). (**C**): Showing slight GR immunostaining in the smooth muscle fibers of the blood vessels of the stratum vascularis (SV) and negative GR immunostaining in the smooth muscle fibers of the inner circular layer (IC) of the myometrium. (**D-H**): Showing slight SOD2 immunostaining in the lamina epithelialis (Ep) and in the stroma cells and the smooth muscle fibers of blood vessels (BV) of the lamina propria (LP) and negative SOD2 immunostaining in the uterine glands (UG). (**I**): Showing slight SOD2 immunostaining in the smooth muscle fibers of blood vessels of stratum vascularis (SV) and negative SOD2 immunostaining in the smooth muscle fibers of the inner circular layer (IC) of the myometrium. Original magnification; (**A, B, C, E, H & I**): X400, scale bar = 50 μm, D, F & G: X200, scale bar = 100 μm

GR immunostaining in the Saidi sheep uterus during the follicular phase of the estrous cycle showed slight GR immunostaining in the lamina epithelialis, and in the stroma cells of lamina propria, and negative GR immunostaining in the uterine glands. Furthermore, slight GR immunostaining in the smooth muscle fibers of the blood vessels of the stratum vascularis and negative GR immunostaining in the smooth muscle fibers of the inner circular layer of the myometrium could be demonstrated (Fig. 4A-C).

Whereas, slight SOD2 immunostaining was observed in the lamina epithelialis, stroma cells, and the smooth muscle fibers of the blood vessels of the lamina propria in addition to no immunostaining of SOD2 in the uterine glands (Fig. 4D-H). Slight SOD2 immunostaining in the smooth muscle fibers of blood vessels of stratum vascularis and negative SOD2 immunostaining in the smooth muscle fibers of the inner circular layer of the myometrium was also noticed (Fig. 4I).

### Apoptosis in the Saidi sheep uterus during the follicular phase of the estrous cycle

**Fig. 5:**
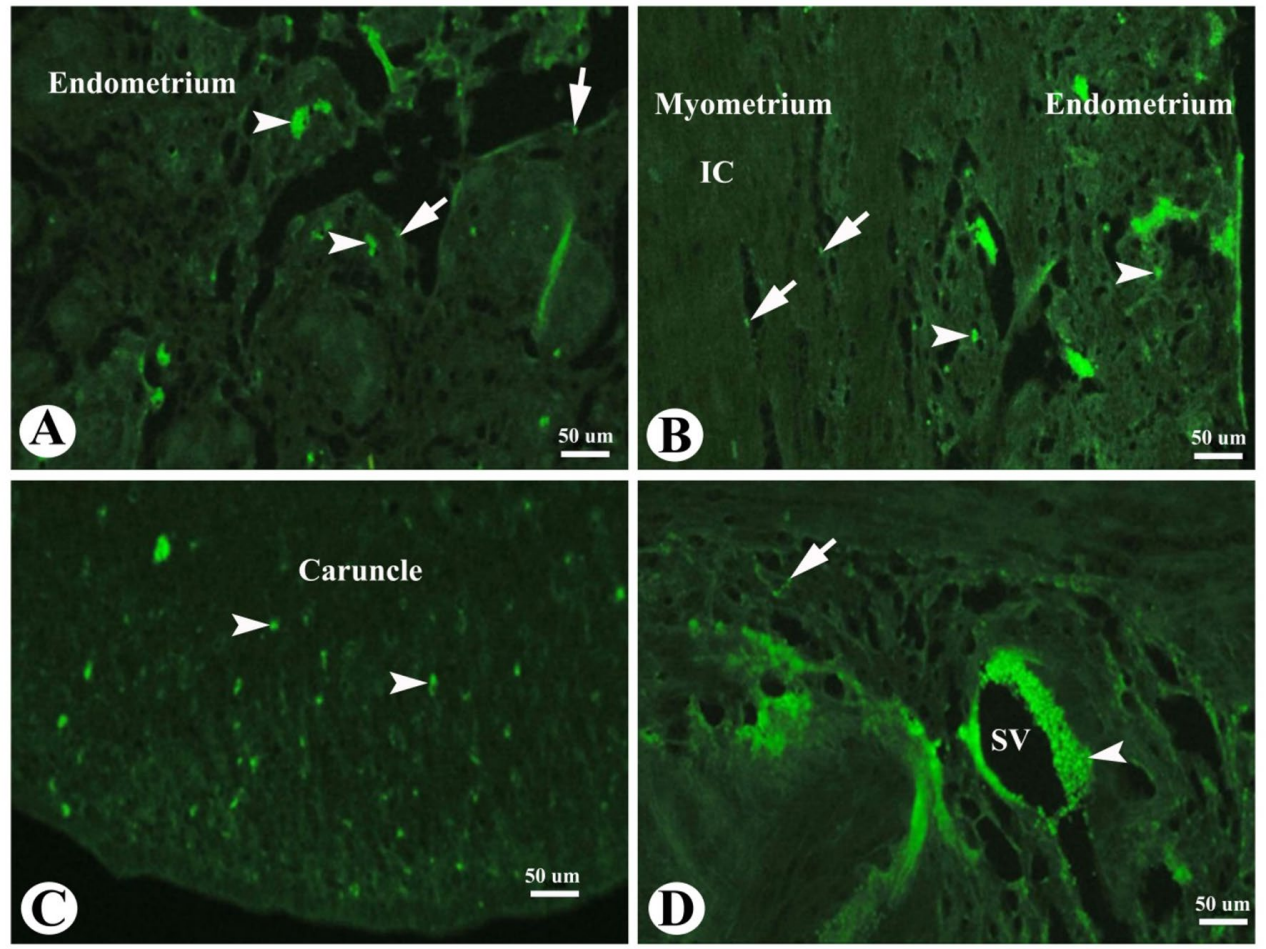
Photomicrograph of TUNEL assay immunofluorescence in the sheep uterus during the follicular phase of estrous cycle. (**A**): Showing apoptotic luminal (arrow) and glandular epithelial cells (arrowheads). (**B**): Showing apoptotic endometrial stromal cells (arrowheads). Note the inner circular layer (IC) of the myometrium with few apoptotic cells (arrow). (**C**): Showing apoptotic endometrial stromal cells (arrowheads) in the caruncle. (**D**): Showing apoptotic smooth muscle fibers (arrowheads) of the blood vessels of the stratum vascularis (SV) and some apoptotic smooth muscle fibers of the myometrium (arrow). Original magnification; (**A-D**): X400, scale bar = 50 μm

TUNEL assay immunofluorescence in the sheep uterus during the follicular phase of estrous cycle explored some apoptotic luminal and glandular epithelial cells (Fig. 5A) and some apoptotic endometrial stromal cells (Fig. 5B). While the inner circular layer of myometrium (IC) showed few apoptotic cells (Fig. 5B). Apoptotic endometrial stromal cells in the caruncles were also observed (Fig. 5C) in addition to apoptotic smooth muscle fibers of the blood vessels of the stratum vascularis (Fig. 5D).

### PRA immunolocalization in the Saidi sheep uterus during the follicular phase of the estrous cycle

**Fig. 6:**
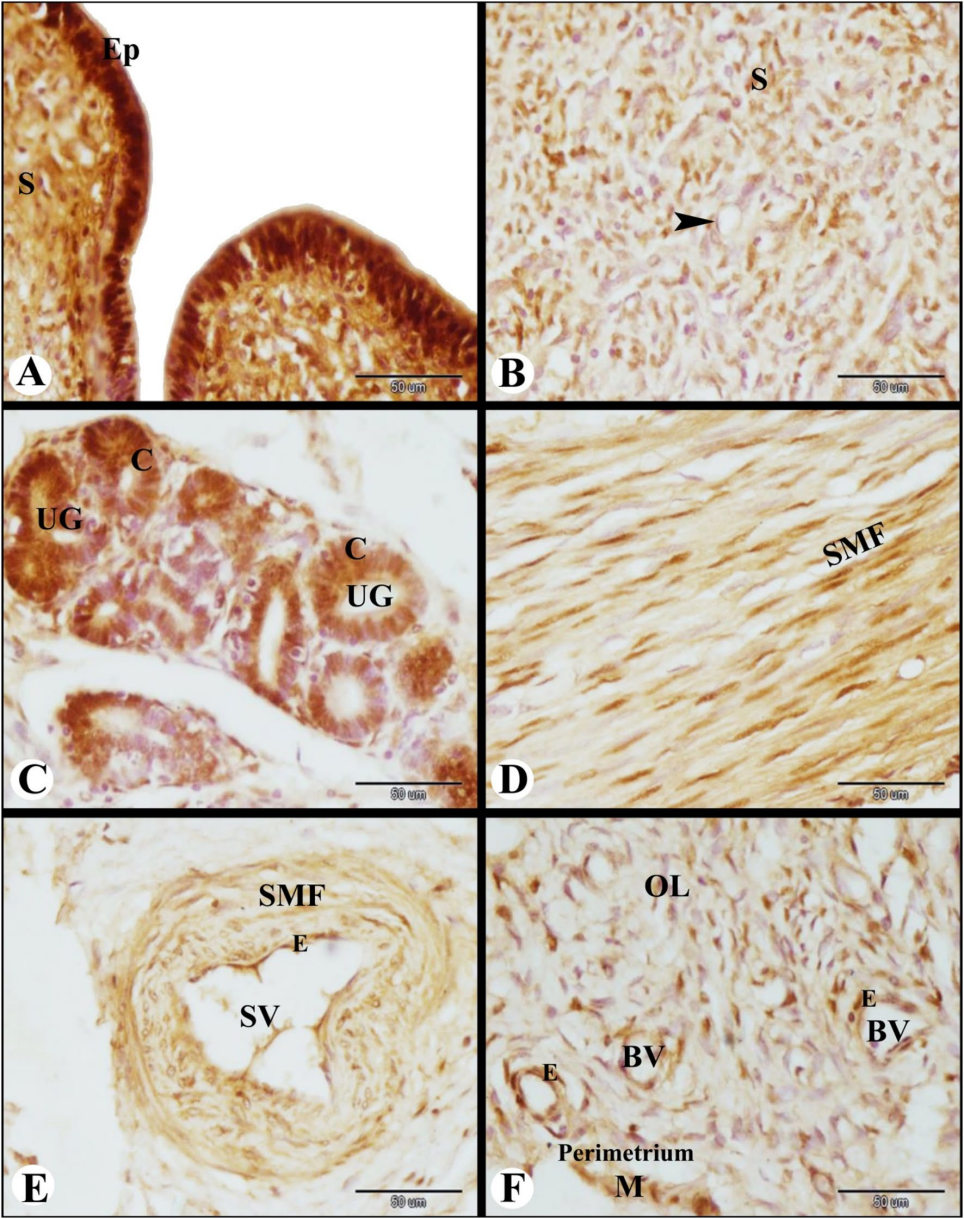
Photomicrograph of PRA immunolocalization in the sheep uterus during the follicular phase of the estrous cycle. (**A**): Showing strong PRA immunolocalization in the lamina epithelialis (Ep) and strong to moderate PRA immunolocalization in the subepithelial stroma cells (S). (**B**): Showing moderate PRA immunolocalization in the stromal cells and endothelial cells (arrowhead) of blood vessels in the caruncles. (**C**): Showing strong to moderate PRA immunolocalization in the columnar epithelial cells (C) of the uterine glands (UG). (**D**): Showing moderate PRA immunolocalization in the smooth muscle fibers (SMF) of the inner circular layer of the myometrium. (**E**): Showing moderate PRA immunolocalization in the endothelium (E) and smooth muscle fibers (SMF) of the blood vessels of the stratum vascularis (SV). (**F**): Showing moderate PRA immunolocalization in the smooth muscle fibers and endothelial cells (E) of blood vessels (BV) in the outer longitudinal layer (OL) of the myometrium and moderate PRA immunolocalization in the mesothelial cells (M) and in the endothelial cells (E) of blood vessels (BV) of the perimetrium. Original magnification; A-F: X400, scale bar = 50 μm

PRA immunolocalization in the Saidi sheep uterus during the follicular phase of the estrous cycle revealed strong PRA immunolocalization in the lamina epithelialis and strong to moderate PRA immunolocalization in the sub-epithelial stroma cells (Fig. 6A). Also moderate PRA immunostaining was observed in the stromal cells and endothelial cells of blood vessels in the caruncles (Fig. 6B). The columnar epithelial cells of the uterine glands expressed strong to moderate PRA immunostaining (Fig. 6C). While the smooth muscle fibers of the inner circular layer of the myometrium expressed moderate PRA immunostaining (Fig. 6D). Moreover, the endothelium and the smooth muscle fibers of the blood vessels of the stratum vascularis showed moderate PRA immunolocalization (Fig. 6E). On the other hand, mild PRA immunoexpression was noticed in the smooth muscle fibers and blood vessel endothelial cells in the myometrium’s outer longitudinal layer. Similarly, there was mild PRA immunolocalization in the perimetrial mesothelial cells, and perimetrial blood vessel endothelial cells (Fig. 6F).

### Mast cells detection in the Saidi sheep uterus during the follicular phase of the estrous cycle

**Fig. 7:**
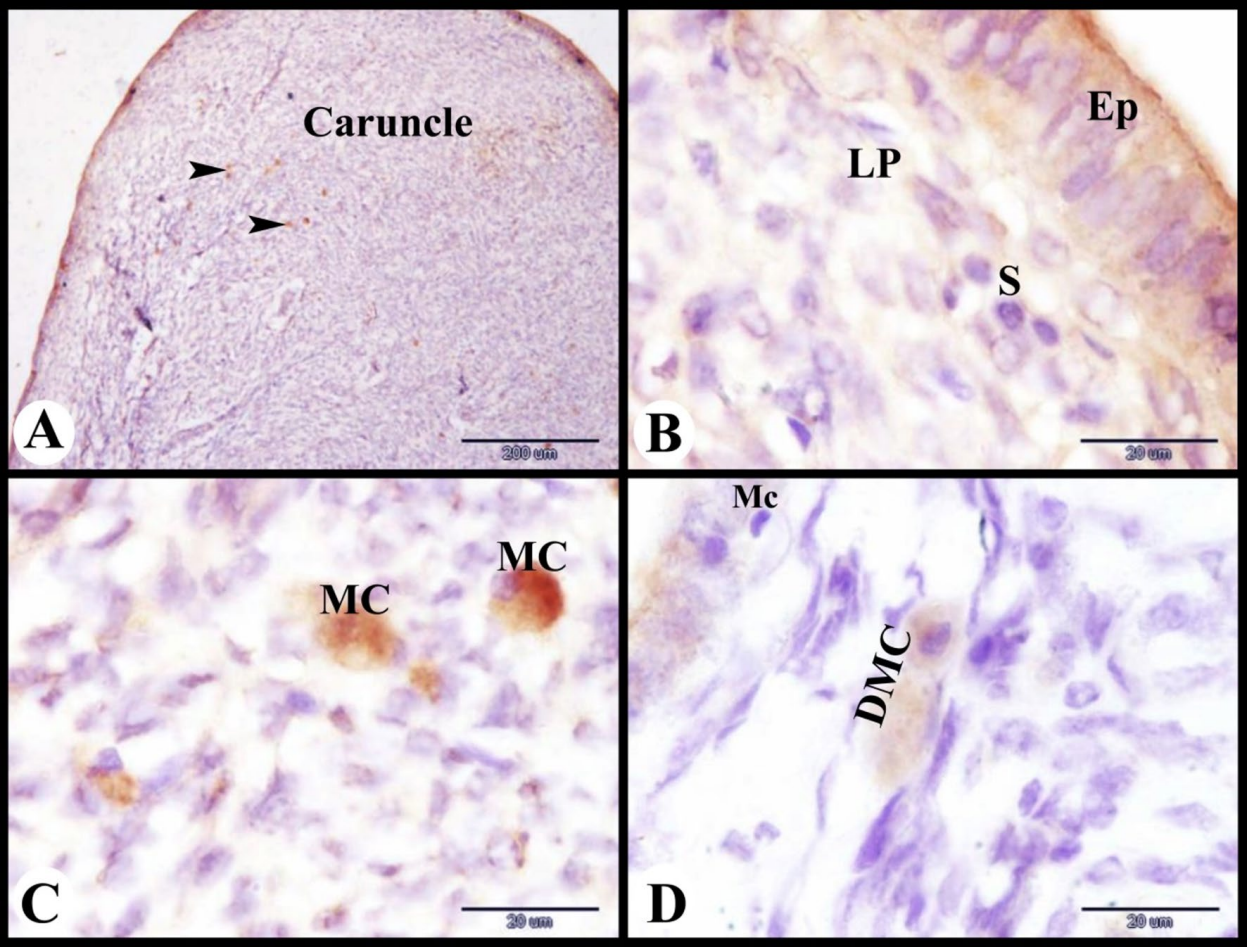
Photomicrograph of mast cells immunolocalization in the sheep uterus during the follicular phase of the estrous cycle. (**A**): Showing tryptase positive immunostaining mast cells (arrowheads) in the deep lamina propria of the caruncles. (**B**): Showing the superficial lamina propria (LP) under the lamina epithelialis (Ep) with stroma cells (S) and no mast cells. (**C**): Showing mast cells (MC) filled with tryptase positive immunostaining granules. (**D**): Showing degranulated mast cells (DMC) near the macrophage (Mc) in the endometrial lamina propria. Original magnification; A: X100, scale bar = 200 μm, B-D: X1000, scale bar = 20 μm

Interestingly, tryptase-positive immunostaining mast cells were recruited in the deep lamina propria of the caruncles during the follicular phase of the estrous cycle (Fig. 7A). Although there were no mast cells seen in the superficial lamina propria underneath the lamina epithelialis (Fig. 7B). Mast cells were rounded or oval cells with rounded central or eccentric nucleus and filled with tryptase positive immunostaining granules (Fig. 7C). Some degranulated mast cells were seen close to the macrophage in the endometrial lamina propria (Fig. 7D).

**Fig. 8:**
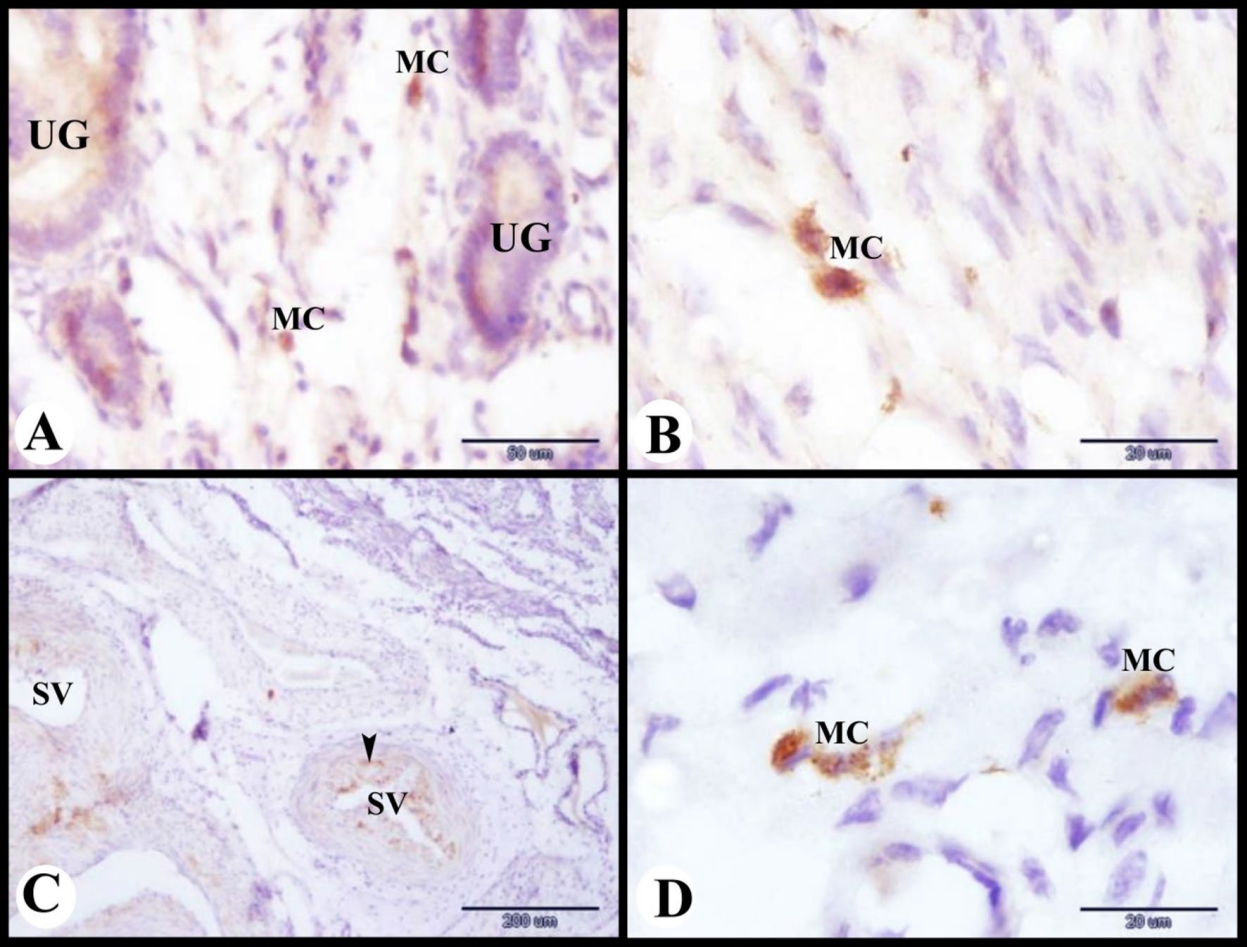
Photomicrograph of mast cells immunolocalization in the sheep uterus during the follicular phase of the estrous cycle. (**A**): Showing tryptase positive immunostaining mast cells (MC) in-between uterine glands (UG). (**B**): Showing tryptase positive immunostaining mast cells (MC) in the inner circular smooth muscle layer of the myometrium. (**C**): Showing tryptase positive immunostaining mast cells (arrowheads) around the blood vessels of the stratum vascularis (SV). (**D**): Showing tryptase positive immunostaining mast cells (MC) in the outer longitudinal smooth muscle layer of the myometrium. Original magnification; A: X400, scale bar = 50 μm, B & D: X1000, Scale bar = 20 μm, C: X100, scale bar = 200 μm

Mast cells were also observed in-between uterine glands (Fig. 8A) and in the inner circular smooth muscle layer of the myometrium (Fig. 8B). Moreover they were noticed in the wall of the blood vessels of the stratum vascularis (Fig. 8C) and in the outer longitudinal smooth muscle layer of the myometrium (Fig. 8D).

**Fig. 9:**
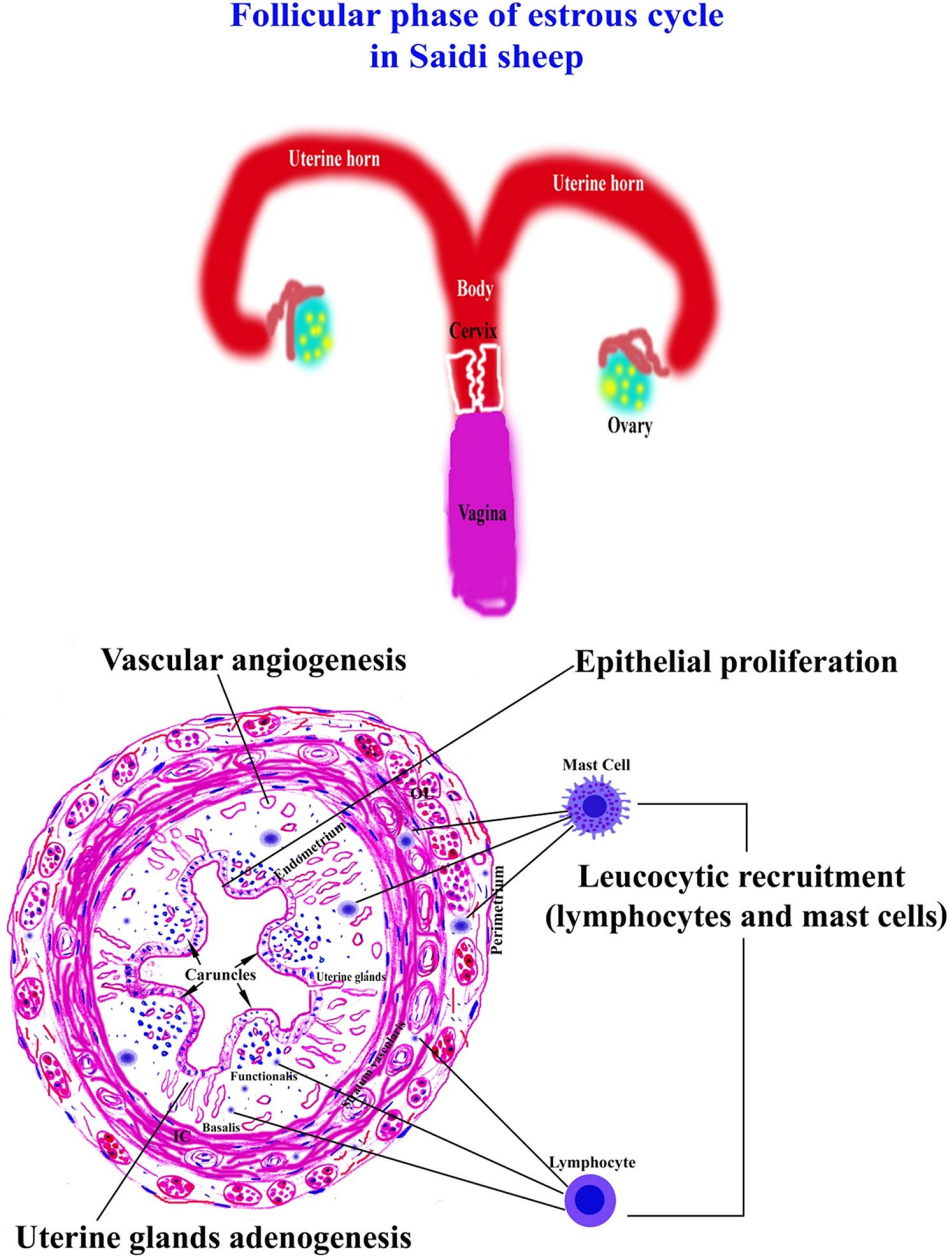
The main uterine characteristics of the follicular phase of the estrous cycle in Saidi sheep

The main uterine characteristics of the follicular phase of the estrous cycle in Saidi sheep are present in Fig. 9 and Table 1.

**Table 1:** Scoring protocol for histological features in the uterus of Saidi sheep during the follicular phase of the estrous cycle

Region	Endometrium			Myometrium		Perimetrium	
Criteria	L.Ep	G.Ep	C.T & BV	SMF	C.T & BV	Meso.	Sub.Meso.
Epithelial proliferation	+	-	-	-	-	-	-
Adenogenesis	**-**	**+**	**-**	**-**	**-**	**-**	**-**
Vascular angiogenesis	**-**	**-**	**+**	-	**+**	**-**	**-**
Glycoprotein	**+BM**	**+BM**	**-**	**-**	**-**	**-**	**-**
Collagen fibers	**-**	**-**	**+**	-	**+**	**-**	**+**
Elastic fibers	**-**	**-**	**+**	-	**+**	**-**	**+**
GR	**+**	**-**	**+**	**-**	**+**	**-**	**-**
SOD2	**+**	**-**	**+**	**-**	**+**	**-**	**-**
Apoptosis (TUNEL)	**+**	**+**	**+**	**+**	**+**	**-**	**-**
PRA	**+**	**+**	**+**	**+**	**+**	**+**	**+**
Mast cells	**-**	**-**	**+**	-	**+**	**-**	**-**

L.Ep: luminal epithelium, G.Ep: glandular epithelium, Meso: mesothelium, sub.Meso: sub mesothelium, BM: basement membrane, C.T: connective tissue, BV: blood vessels

## Discussion

Microscopical analysis of the uterus of Saidi sheep during the follicular phase of the estrous cycle revealed that the uterine wall was composed of three layers: the inner endometrium, middle myometrium, and the outer perimetrium. The endometrium had mucosal folds, caruncles, and narrow uterine lumen. The endometrium formed of lamina epithelialis of pseudostratified columnar epithelium with intraepithelial lymphocytes and connective tissue lamina propria contained uterine glands, fibroblasts, collagen fibers, blood vessels, and leucocytic infiltrations, especially lymphocytes and plasma cells. The uterus (horns and body) in ruminants was lined with simple columnar to pseudostratified columnar epithelium and the mean height of the epithelium was less in follicular phase [16, 35, 36]. In buffalo the endometrium was lined with three types of columnar cells, i.e. ciliated, non-ciliated cells, and basal cells [36]. The endometrial stroma (propria submucosa) consisted of fibro-reticular connective tissue, stromal cells, and blood vessels. Its cellular components comprise stromal cells, fibroblasts, mesenchymal cells, neutrophils, and lymphocytes. The stromal cells’ nuclei were elliptical, oval, or circular in shape. In the follicular phase, the stroma was very crowded and swollen [16, 36]. The endometrium showed a period of growth preceded by vascularization during the follicular phase of the estrous cycle in sheep [37]. Leukocytes invaded the functional layer on Day 7 of the estrous cycle in cows [38]. The bovine luminal epithelium changes during the estrous cycle through a remodeling process [39].

The endometrium in adult ruminants (sheep, goat, buffalo and cattle) consists of aglandular caruncles and glandular intercaruncular areas [10]. The caruncles were non glandular, highly cellular, and highly vascular endometrial elevation or projections. The locations of superficial implantation and placentation occur in caruncular regions. Interdigitation and branching morphogenetic growth of placental cotyledons with endometrial caruncles create placentomes in synepitheliochorial placentation observed in ruminants. Placentomes are primarily involved in fetal-maternal gas exchange and the placenta’s absorption of micronutrients for hemotrophic nutrition of the fetus [10].

The current study showed that the follicular phase of estrous cycle in Saidi sheep was characterized by epithelial proliferation and many epithelial invaginations which form the uterine glands (uterine gland adenogenesis). Herein, the uterine glands were highly branched, highly coiled, and formed of columnar epithelium surrounded by myoepithelial cells and had intraepithelial lymphocytes. Similar results were obtained in goats during the follicular phase of the estrous cycle [16, 35]. While uterine glands in sheep and pigs are tightly coiled, heavily branched tubular glands, uterine glands in mice are comparatively simple tubes with little branching [40]. These glands have occasionally penetrated and reached the stratum vascularis. Proliferation of the endometrial glands in the follicular phase [35] was as a result of glandular epithelium mitoses [38].

Uterine gland adenogenesis, is uniquely a postnatal event in sheep and pigs and involves differentiation and budding of glandular epithelium from luminal epithelium, followed by invagination and extensive tubular coiling and branching morphogenesis throughout the uterine stroma to the myometrium. Uterine adenogenesis is regulated by both intrinsic transcription factors and extrinsic factors from the pituitary, ovary, and mammary gland (lactocrine) [14, 41]. It was found that prolactin, estradiol-17b, and their receptors are implicated in mechanisms controlling endometrial adenogenesis in many animals and humans [14].

The process of uterine vascular remodeling and angiogenesis is strictly regulated. It is important to the cycling and early pregnant endometrium. The endometrium and the macrophages that reside there can produce most of the key cytokines and factors that are currently known to be involved in the regulation of angiogenesis [42]. Some of these factors expressed throughout the menstrual cycle include vascular endothelial growth factor (VEGF) [43, 44], fibroblast growth factor (FGF) [45] transforming growth factor-α (TGF-α) [46], interleukin (IL)-1 and IL-6 [47], epidermal growth factor (EGF) [48], IL-8 [49] and ovarian hormones, angiopoietins, Notch, and uterine natural killer cells [17].

The current study showed that few elastic fibers were observed in the connective tissue of the endometrium and myometrium. Similar findings were observed in humans [50] and mice [51]. The uterine elasticity is likely maintained without excess stress being placed on the developing fetus by these thin sheets of elastic membranes and elastic fibrils [52]. Elastic fibers are resistant to tensile stresses and have persistently variable functions based on the needs of the microenvironment in which they are found [53].

Our findings revealed that the collagen fibers were more thickly distributed in the lamina propria of the uterine endometrium close to the endometrial glands and were located between the muscles [53]. While the intercellular matrix of the endometrial stroma contained a moderate amount of collagen fibers [36]. It has been suggested that the variability of the connective tissue thread distribution in the uterus may have a role in the fertilization process [53]. Because collagen fibers are found in the stroma and between the muscles, they enable the uterus to contract and stretch [53]. Collagen fibers visualization could make it easier to assess the thickness of the connective tissue that envelops endometrial glands. Elevated density may lead to degeneration and loss of function by impairing the flow of nutrients and endocrine signaling molecules from blood arteries to the glandular epithelium [54].

Our results showed slight GR & SOD2 immunostaining in the lamina epithelialis, the stroma cells of lamina propria, and the smooth muscle fibers of the blood vessels of the stratum vascularis. Estradiol and progesterone control uterine glutathione reductase, which may be crucial in preserving the uterus’s lowered glutathione levels. This molecule may be necessary for detoxification reactions involving H2O2 and electrophylic chemicals as well as for the regulation of the redox state of thiol groups. Glutathione reductase is stimulated by estrogens, which contributes to their antioxidant properties [25]. The uterus and fallopian tube contain antioxidants that aid in removing excess reactive oxygen species (ROS), creating the ideal environment for embryonic growth. To get rid of the ROS that cytokines and inflammation produce in mitochondria, SOD2 content is raised [26]. In addition, there was a close relation between increased ROS and apoptosis. SOD2 and GR expression help to control apoptosis during the estrous cycle [5].

Our finding by using TUNEL assay indicated that apoptosis is necessary for uterine remodeling during the follicular phase of estrous cycle in Saidi sheep. The uterine epithelium of rats and hamsters has been shown to undergo apoptosis throughout the estrous cycle. During the estrous cycle, rat uterine and vaginal epithelial cells showed an inverse relationship between cell death and proliferation. Uterine epithelial cell proliferation, differentiation, and death are regulated by estrogen and progesterone [23, 24]. In human uterus, apoptotic uterine cells are scattered in the functional layer of the early proliferative endometrium [24]. Homeostasis of the uterus is closely related to apoptosis and involves many hormones and cytokines [55]. Our results indicate that apoptosis might have a crucial role in regulating the estrous cycle in Saidi sheep.

The current study revealed PRA immunolocalization in the lamina epithelialis, stroma cells, endothelial cells, columnar epithelial cells of the uterine glands and in the smooth muscle fibers of the myometrium. Moreover, the endothelium and the smooth muscle fibers of the blood vessels of the stratum vascularis showed also PRA immunolocalization. On the other hand, there was PRA immunolocalization in the perimetrial mesothelial cells and perimetrial endothelial cells. Similar findings were found in rabbit uterus during pseudopregnancy [9]. Progesterone, a critical steroid hormone in the reproductive system, plays a vital role during the follicular phase of the estrous cycle [56]. Although traditionally associated with the luteal phase, emerging research highlights its significant functions in the follicular phase [57]. Progesterone receptors (PRs) exist in two main isoforms, PR-A and PR-B, and are differentially expressed in ovarian follicles and uterus [58]. Their expression is dynamically regulated by fluctuating hormone levels throughout the cycle. Progesterone via acting through PR-A is also essential for uterine decidualization and implantation [58]. PRA is mainly a nuclear receptor but may be expressed in the cytoplasm. During the proliferative phase of the human uterus, PR were increased mainly in the cytoplasm [59]. While PRA was expressed in both the nucleus and cytoplasm in the rabbit ovary during pregnancy and pseudopregnancy [34, 60] and in the sheep ovary during follicular phase of estrous cycle [5]. In mare, steroid hormone receptors showed greater expression in the estrus phase and a lower expression during the diestrus phase, this was due to estrogen stimulation that elevated the expression of progesterone receptors [61–63].

It was discovered that the plane of nutrition, the estrous cycle phase, and/or FSH all had an impact on the percentage of PR-positive uterine cells and/or staining intensity [64]. Through a complicated paracrine signaling network, progesterone through binding to the PR controls glandular growth, decidualization, implantation, and maintenance of a healthy uterus [65–67].

Interestingly, tryptase-positive immunostaining mast cells were recruited in the uterine connective tissue and around the blood vessels of the stratum vascularis. MCs during the follicular phase of the estrous cycle play a crucial role in secreting substances that promote tissue remodeling [21]. Histamine is one of these important substances released from uterine mast cells, influencing ovulation, embryo implantation, and myometrium contractility leading to successful implantation and ultimately to parturition [68–70]. However, spatiotemporally expression of MCs in the female reproductive tract has been reported in other studies [70–74]. In the uterus, they change in number and structure depending on the hormonal level variations during the menstrual or estrous cycle [75–78]. A previous study has revealed alterations in the characteristics and location of MCs in mice, rats, hamsters, cows, and guinea pigs uterine tissues during pregnancy and estrous cycle [79, 80]. Notably, the number and activity of MCs are correlated with estrogen concentrations in the uterine tissue of sows and rats [81, 82], while they are correlated with progesterone concentrations in the canine uterus [70].

Nevertheless, to preserve regular reproductive processes and create the best environment for potential implantation, all uterine cell types interact with one another through junctacrine, paracrine, or endocrine pathways [64].

## Conclusion

This study provides new evidence of the role of PRA, SOD2, GR, and mast cells in controlling uterine epithelial proliferation and apoptosis in the Saidi sheep during the follicular phase of the estrus cycle. These findings have growing significance in understanding the key mechanisms that characterize successful reproduction in Saidi sheep to enhance the fertility and reproductive health of this livestock species and help to use advanced reproductive techniques.

## Data Availability

The datasets used and/or analysed during the current study are available from the corresponding author on reasonable request.

## Abbreviations

PBS: Phosphate buffered saline
GR: Glutathione reductase
SOD2: Superoxide dismutase 2
TUNEL: Terminal deoxynucleotidyl transferase (TdT) dUTP Nick-End Labeling
PRA: Progesterone receptor alpha
PRB: Progesterone receptor beta
PAS: Periodic acid Schiff
VEGF: Vascular endothelial growth factor
FGF: Fibroblast growth factor
TGF-α: Transforming growth factor-α
IL: Interleukin
EGF: Epidermal growth factor
PGF2α: Prostaglandin F2α
PGE2: Prostaglandin E2
FSH: Follicle-stimulating hormone
LH: Luteinizing hormone
FSHR: Follicle-stimulating hormone receptor
MMPs: Matrix metalloproteinases
Mast cells: MCs
ROS: Reactive oxygen species
